# Supplementary material for: Ten years of graduates: A cross-sectional study of the practice location of doctors trained at a socially accountable medical school
Source: PLoS One. 2022 Sep 15;17(9):e0274499. doi: 10.1371/journal.pone.0274499 (PMC9477294; doi:10.1371/journal.pone.0274499)
Supplement: S1 Table — (DOCX) [file pone.0274499.s001.docx]

**Table S1. List of specialties by specialty group.**

| **Specialty Group** | **Count** |
| --- | --- |
| *Family Medicine (PG offered at NOSM or at other medical schools)* | |
| Family Medicine (FM) | 283 |
| Family Medicine and a FM PGY3 * | 51 |
| *Subtotal* | *334* |
| *Generalist Specialists (PG offered at NOSM or at other medical schools)* | |
| Pediatrics | 16 |
| Orthopedic Surgery | 10 |
| Obstetrics and Gynecology | 9 |
| Anesthesiology | 8 |
| Psychiatry | 7 |
| General Surgery | 6 |
| Internal Medicine | 5 |
| Public Health & Preventative Medicine/Community Medicine | 1 |
| *Subtotal* | *62* |
| *All other Specialists (PG only offered at other medical schools)* | |
| Internal Medicine subspecialty: Clinical Immunology & Allergy; Critical Care Medicine; Endocrinology & Metabolism; Geriatrics; Infectious Diseases; Medical Oncology; Respirology; and Rheumatology | 9 |
| Pediatric subspecialty: Clinical Immunology & Allergy; Emergency Medicine; Endocrinology; and Respiratory Medicine | 6 |
| Psychiatry subspecialty: Child & Adolescent;† and Geriatrics | 5 |
| Dermatology | 4 |
| Emergency Medicine | 3 |
| Radiation subspecialty: Radiation Oncology; and Diagnostic Radiology | 3 |
| Otolaryngology | 2 |
| Plastic Surgery | 2 |
| Thoracic (Cardiac) Surgery | 2 |
| Pathology subspecialty: Anatomical & General Pathology; and Hematological Pathology | 2 |
| Ophthalmology | 1 |
| *Subtotal* | *39* |
| **Grand Total** | **435** |

Note: NOSM = Northern Ontario School of Medicine.

* These doctors completed their UG, PG, or both at NOSM before completing their FM PGY3 at NOSM. Doctors who only completed a FM PGY3 at NOSM were excluded. Thirty in this group had completed a FM in an integrated program in Anesthesiology, Emergency Medicine, Obstetrics, or Palliative Care.

† Child & Adolescent Psychiatry can also be considered as a pediatric subspecialty.

[Hogenbirk et al. 2022. PLOS ONE]
