# Supplementary material for: Ten years of graduates: A cross-sectional study of the practice location of doctors trained at a socially accountable medical school
Source: PLoS One. 2022 Sep 15;17(9):e0274499. doi: 10.1371/journal.pone.0274499 (PMC9477294; doi:10.1371/journal.pone.0274499)
Supplement: S2 Table — (DOCX) [file pone.0274499.s002.docx]

**Count of cases excluded from analyses and comparison with included cases.**

Cases were excluded because they either lack comparison groups (e.g., International medical graduates can only belong to the NOSM PG group), had a mix of practice locations (e.g., locum tenens, additional training), or had only minor exposure to NOSM medical education (e.g., PGY 3 only) (**Table S2 Part A**). Other cases were excluded because they lacked data for the logistic regression models.

**Table S2 Part A. Cases excluded from analyses.**

| Count of cases | Explanation |
| --- | --- |
| **692** | Number of valid cases of doctors who entered undergraduate medical education in 2005 or later and had completed all residency training. These doctors have some or all of their medical education or residency training at the Northern Ontario School of Medicine (NOSM). |
| 97 | International medical graduates (IMGs) were excluded because they train outside of Canada for their medical degree and come to the NOSM only for their postgraduate training. In this data set, IMGs can only belong to the NOSM PG group. |
| 77 | These doctors were excluded because they have only been in full practice for less than one year. Doctors in this group tend to do locum tenens, take time off, or pursue additional training, and therefore their first year practice location may be in flux. An additional 12 doctors with one year or less of full practice were already excluded because they were IMGs. |
| 27 | These doctors were excluded because they came to NOSM only to complete family medicine PGY3 training. An additional seven doctors were already excluded because they were either IMGs or had less than one year in full practice. |
| 56 | These doctors were excluded because they had missing data for age, hometown, or practice location, which, in addition to variables on gender, UG/PG path and specialty group, were needed for the logistic regression analyses. |
| **435** | **Total included in logistic regression models** |

**Comparison of excluded and included cases.**

A comparison of excluded and included cases did not find any significant differences in age at PG entry or age in 2019 (n=44, t-test, p>0.09), percent female, percent with Indigenous status, percent in different specialty groups, percent with a service contract, percent having rural Canada hometown, and percent having rural Canada practice location (Fisher’s exact test, 2-sided, p>0.13) (**Table S2 Part B**).

A lower percentage of the doctors who were excluded from analysis had French language ability (22% versus 38%, p=0.02) and a higher percentage had completed their undergraduate medical degree at another medical school before coming to NOSM for their postgraduate residency training, with lower percentages in the other educational paths (p<0.001). Lower percentages of excluded doctors had a Northern Ontario hometown (39% versus 73%, p=0.003), or had a Northern Ontario practice location (34% versus 54%, p=0.02). The effect of these cases with missing data on the logistic regression model predicting a Northern Ontario practice location was unknown. However, the effect on the logistic regression model predicting a rural Canada practice location was considered to be minimal because a lack of differences between excluded and included cases for a rural Canada hometown and rural Canada practice location.

**Table S2 Part B Excluded (maximum n=56) and included (maximum n=435) cases compared for differences in demographic attributes, UG-PG path, specialty group, and outcomes.**

|  | Age at postgraduate entry (years) | Age in 2019 (years) |
| --- | --- | --- |
| *Count of excluded cases with data* | 44 | 44 |
| *excluded* | 30.4 | 38.6 |
| *included* | 30.2 | 37.1 |
| *p=** | 0.89 | 0.10 |

|  | Gender (female) | Indigenous status (yes) † | French language ability (yes) ‡ | Have a service contract (yes) § |
| --- | --- | --- | --- | --- |
| *Count of excluded cases with data* | 56 | 22 | 55 | 12 |
| *excluded* | 57% | 18% | 22% | 17% |
| *included* | 68% | 9% | 38% | 24% |
| *p=*** | 0.13 | 0.25 | 0.02 | 0.74 |

|  | Northern Ontario hometown | Rural Canada hometown | Outcome: Northern Ontario practice location | Outcome: Rural Canada practice location |
| --- | --- | --- | --- | --- |
| *Count of excluded cases with data* | 18 | 17 | 41 | 41 |
| *excluded* | 39% | 41% | 34% | 17% |
| *included* | 73% | 32% | 54% | 22% |
| *p=*** | 0.003 | 0.43 | 0.02 | 0.56 |

|  | UG-PG path | Specialty group |
| --- | --- | --- |
| *Count of excluded cases with data* | 56 | 56 |
| *excluded doctors:* | Fewer are NOSM UG/NOSM PG |  |
|  | Fewer are NOSM UG/other PG |  |
|  | More are other UG/NOSM PG |  |
| *p=*** | <0.001 | 0.78 |

* Probability for a t-test with no assumption of equal variances.

† 418 included cases

‡ 434 included cases

§ 392 included cases

** Probability for Fisher’s exact test, two sided. For some comparisons, the probability was generated from a Monte Carlo simulation based on 10,000 tables randomly created from the observed table.

[Hogenbirk et al. 2022. PLOS ONE]
