# Supplementary material for: Ten years of graduates: A cross-sectional study of the practice location of doctors trained at a socially accountable medical school
Source: PLoS One. 2022 Sep 15;17(9):e0274499. doi: 10.1371/journal.pone.0274499 (PMC9477294; doi:10.1371/journal.pone.0274499)
Supplement: S3 Table — (DOCX) [file pone.0274499.s003.docx]

**Bivariate association between hometown region and practice region.**

Cross-tabulations, used to explore significant predictors identified in the logistic regressions models, found that FPs with a Northern Ontario hometown had statistically significant (p<0.04) unadjusted ORs of 4.9 – 5.8 of practising in Northern Ontario for all UG-PG paths (**Table S3**). There was no evidence of a similar relationship (p≥0.16) for any non-family medicine specialty group.

**Table S3. Northern Ontario hometown location by Northern Ontario practice location by UG-PG path for each specialty group.***

|  | **UG-PG Path** | **Northern Ontario Hometown?** † | | **Northern Ontario practice location?** | | **Total** | |
| --- | --- | --- | --- | --- | --- | --- | --- |
|  |  |  |  | **Yes** | **No** |  |  |
| **(a) Family Medicine (PG offered at NOSM or at other medical schools)** | | | | | | | |
| Exact p=0.04  OR=4.9, (95% CI=1.1 - 21.9), p=0.04 | *NOSM-Other* | **Yes** | Count | 28 | 72 | **100** | |
|  |  |  | % across row | 28.0 | 72.0 | **100** | |
|  |  | **No** | Count | 2 | 25 | **27** | |
|  |  |  | % across row | 7.4 | 92.6 | **100** | |
|  |  | **Total** | **Count** | **30** | **97** | **127** | |
|  |  |  | **% across row** | **23.6** | **76.4** | **100** | |
| Exact p=0.02  OR=5.5, (95% CI=1.4 - 21.6), p=0.01 | *NOSM-NOSM* | **Yes** | Count | 116 | 7 | **123** | |
|  |  |  | % across row | 94.3 | 5.7 | **100** | |
|  |  | **No** | Count | 12 | 4 | **16** | |
|  |  |  | % across row | 75.0 | 25.0 | **100** | |
|  |  | **Total** | **Count** | **128** | **11** | **139** | |
|  |  |  | **% across row** | **92.1** | **7.9** | **100** | |
| Exact p=0.01  OR=5.8, (95% CI=1.5 – 22.4), p=0.01 | *Other-NOSM* | **Yes** | Count | 19 | 3 | **22** | |
|  |  |  | % across row | 86.4 | 13.6 | **100** | |
|  |  | **No** | Count | 24 | 22 | **46** | |
|  |  |  | % across row | 52.2 | 47.8 | **100** | |
|  |  | **Total** | **Count** | **43** | **25** | **68** | |
|  |  |  | **% across row** | **63.2** | **36.8** | **100** | |
| **Total for Family Medicine**  Exact p<0.001  OR=2.7, (95% CI=1.6 - 4.4), p<0.001 | ***All UG-PG paths*** | **Yes** | Count | 163 | 82 | **245** | |
|  |  |  | % across row | 66.5 | 33.5 | **100** | |
|  |  | **No** | Count | 38 | 51 | **89** | |
|  |  |  | % across row | 42.7 | 57.3 | **100** | |
|  |  | **Total** | **Count** | **201** | **133** | **334** | |
|  |  |  | **% across row** | **60.2** | **39.8** | **100** | |
| **(b) Generalist Specialties (PG offered at NOSM or at other medical schools)**‡ | | | | | | |  |
| Exact p=0.17  OR=2.5, (95% CI=0.8 - 8.1), p=0.13 | ***All UG-PG paths*** | **Yes** | Count | 18 | 23 | **41** |  |
|  |  |  | % across row | 43.9 | 56.1 | **100** |  |
|  |  | **No** | Count | 5 | 16 | **21** |  |
|  |  |  | % across row | 23.8 | 76.2 | **100** |  |
|  |  | **Total** | **Count** | **23** | **39** | **62** |  |
|  |  |  | **% across row** | **37.1** | **62.9** | **100** |  |

|  | **UG-PG Path** | **Northern Ontario Hometown?** † | | **Northern Ontario practice location?** | | **Total** | |
| --- | --- | --- | --- | --- | --- | --- | --- |
|  |  |  |  | **Yes** | **No** |  |  |
| **(c) All Other Specialties (PG only offered at other medical schools)**‡ | | | | | | |  |
| Exact p=0.16  OR could not be computed | ***All UG-PG paths*** | **Yes** | Count | 9 | 22 | **31** |  |
|  |  |  | % across row | 29.0 | 71.0 | **100** |  |
|  |  | **No** | Count | 0 | 8 | **8** |  |
|  |  |  | % across row | 0.0 | 100 | **100** |  |
|  |  | **Total** | **Count** | **9** | **30** | **39** |  |
|  |  |  | **% across row** | **23.1** | **76.9** | **100** |  |

Note: Exact p: probability calculated with Fisher’s Exact test, 2-sided, NOSM: Northern Ontario School of Medicine, OR: (unadjusted) odds ratio, with asymptotic 95% confidence intervals (CI) and probability, Other: Other medical school (not NOSM), UG-PG path: Undergraduate medical education school-Postgraduate residency training school.

* Specialty group: refer to **S1 Table** for list of specialties by group.

† See Figure 1 for NOSM’s service region in Northern Ontario. Hometown is where the doctor lived for at least nine years from birth to 18 years of age.

‡ Non-family medicine specialties were collapsed across UG-PG path because there were insufficient numbers and/or little variation among paths.

[Hogenbirk et al. 2022. PLOS ONE]
