# Supplementary material for: Ten years of graduates: A cross-sectional study of the practice location of doctors trained at a socially accountable medical school
Source: PLoS One. 2022 Sep 15;17(9):e0274499. doi: 10.1371/journal.pone.0274499 (PMC9477294; doi:10.1371/journal.pone.0274499)
Supplement: S4 Table — (DOCX) [file pone.0274499.s004.docx]

**Supplement 5:** Bivariate association between hometown rurality and practice rurality.

Having a rural hometown had a statistically significant (p<0.001) unadjusted OR of 5.5 (95% CI= 2.4 – 12.6) for FPs in rural practice, but only for the NOSM UG/NOSM PG path (**Table S4**). For generalist specialists, significantly more doctors with a rural hometown practised in rural Canada (3/17=18%) compared with doctors from an urban hometown (0/45=0%) (p=0.02). There was no similar association for other NON-FAMILY MEDICINE specialists (p=0.38).

**Table S4. Rural hometown location by rural practice location by UG-PG path for each specialty group.***

|  | **UG-PG Path** | **Rural Hometown?** † | | **Rural practice location?** | | **Total** |
| --- | --- | --- | --- | --- | --- | --- |
|  |  |  |  | **Yes** | **No** |  |
| **(a) Family Medicine (PG offered at NOSM or at other medical schools)** | | | | | | |
| Exact p=0.43  OR=1.4, (95% CI=0.6 - 3.0), p=0.42 | *NOSM-Other* | Yes | Count | 17 | 39 | **56** |
|  |  |  | % across row | 30.4 | 69.6 | **100** |
|  |  | No | Count | 17 | 54 | **71** |
|  |  |  | % across row | 23.9 | 76.1 | **100** |
|  |  | **Total** | **Count** | **34** | **93** | **127** |
|  |  |  | **% across row** | **26.8** | **73.2** | **100** |
| Exact p=<0.001  OR=5.5, (95% CI=2.4 – 12.6), p=<0.001 | *NOSM-NOSM* | Yes | Count | 20 | 19 | **39** |
|  |  |  | % across row | 51.3 | 48.7 | **100** |
|  |  | No | Count | 16 | 84 | **100** |
|  |  |  | % across row | 16.0 | 84.0 | **100** |
|  |  | **Total** | **Count** | **36** | **103** | **139** |
|  |  |  | **% across row** | **25.9** | **74.1** | **100** |
| Exact p=1.00  OR=1.1, (95% CI=0.3 - 4.4), p=0.85 | *Other-NOSM* | Yes | Count | 4 | 7 | **11** |
|  |  |  | % across row | 36.4 | 63.6 | **100** |
|  |  | No | Count | 19 | 38 | **57** |
|  |  |  | % across row | 33.3 | 66.7 | **100** |
|  |  | **Total** | **Count** | **23** | **45** | **68** |
|  |  |  | **% across row** | **33.8** | **66.2** | **100** |
| Exact p=0.004  OR=2.1, (95% CI=1.3 - 3.5), p=0.003 | ***All UG-PG paths*** | Yes | Count | 41 | 65 | **106** |
|  |  |  | % across row | 38.7 | 61.3 | **100** |
|  |  | No | Count | 52 | 176 | **228** |
|  |  |  | % across row | 22.8 | 77.2 | **100** |
|  |  | **Total** | **Count** | **93** | **241** | **334** |
|  |  |  | **% across row** | **27.8** | **72.2** | **100** |
| **(b) Generalist Specialties (at NOSM or at other medical schools)**‡ | | | | | | |
| Exact p=0.02  OR could not be computed | *all UG-PG paths* | Yes | Count | 3 | 14 | **17** |
|  |  |  | % across row | 17.6 | 82.4 | **100** |
|  |  | No | Count | 0 | 45 | **45** |
|  |  |  | % across row | 0.0 | 100 | **100** |
|  |  | **Total** | **Count** | **3** | **59** | **62** |
|  |  |  | **% across row** | **4.8** | **95.2** | **100** |
| **(c) All other Specialties (only at other medical schools)** ‡ | | | | | | |
| Exact p=0.38  OR could not be computed | *all UG-PG paths* | Yes | Count | 1 | 14 | **15** |
|  |  |  | % across row | 6.7 | 93.3 | **100** |
|  |  | No | Count | 0 | 24 | **24** |
|  |  |  | % across row | 0.0 | 100 | **100** |
|  |  | **Total** | **Count** | **1** | **38** | **39** |
|  |  |  | **% across row** | **2.6** | **97.4** | **100** |

Note: Exact p: probability calculated with Fisher’s Exact test, 2-sided, NOSM: Northern Ontario School of Medicine, OR: (unadjusted) odds ratio, with asymptotic 95% confidence intervals (CI) and probability, Other: Other medical school (not NOSM), UG-PG path: Undergraduate medical education school-Postgraduate residency training school.

* Specialty group: refer to **S1 Table** for list of specialties by group.

† Rural was defined as a census subdivision of less than 10,000 people that was located outside of a census metropolitan area or outside of a census agglomeration (Statistics Canada 2003). Hometown is where the doctor lived for at least nine years from birth to 18 years of age.

‡ Non-family medicine specialties were collapsed across UG-PG path because there were insufficient numbers and/or little variation among paths.

[Hogenbirk et al. 2022. PLOS ONE]
