# Supplementary material for: Ten years of graduates: A cross-sectional study of the practice location of doctors trained at a socially accountable medical school
Source: PLoS One. 2022 Sep 15;17(9):e0274499. doi: 10.1371/journal.pone.0274499 (PMC9477294; doi:10.1371/journal.pone.0274499)
Supplement: Attachment — Submitted filename: Comments.docx [file pone.0274499.s005.docx]

Comments/Questions

Abstract: some of the strongest data, in my opinion, is the aOR of 45 for FPs practicing in northern Ontario who completed UG and PG at NOSM vs. rural background aOR = 5 Would recommend adding this to the abstract

Line 21	We used a cross-sectional study to measured

Is the word “measured” necessary? It first appears to be a typo (e.g. to measure)

Line 47-48 NOSM selects students from underserved and economically deprived regions of Canada with a focus on northern Ontario

This can be strengthened with some data, such as “____% of matriculating students at NOSM are from….”

Line 60 typo	“with most of the these”

Lines 269-270 . Completing both UG and PG at NOSM had the highest aOR of 45.7

Since there is a lot of data, would recommend qualifying this statement, Completing both UG and PG at NOSM had the highest aOR of 45.7 for FPs practising in northern Ontario.

Line 280-282	The overall percentage was nearly twice that 280 of the 49% (508/1047) of doctors who graduated 1973 – 2008 from the Memorial University of 281 Newfoundland (MUN) and did at least some of their PG training at MUN and were practising in MUN’s service region in 2014.[27]

Intriguing. A very nice comparator. For those not familiar, what is the rationale for using MUN as a comparator? Are they similarly situated?

Lines 326-334 seem out of place for this manuscript

Line 347	It’s fair to say the following, yes? The second, and more influential factor in our study, is to

348 provide undergraduate and postgraduate medical education immersed in the regions, whereby students 349 and trainees are living and learning in the community and in the clinical settings where they are 350 expected to practice in the future.

Many are going to be interested in this conclusion because while we recognize the value of admitting students from rural backgrounds into medical school, and we recognize the role of the medical school educational environment, we do not know which is more influential. This study appears to help answer that.
