# Supplementary material for: Ten years of graduates: A cross-sectional study of the practice location of doctors trained at a socially accountable medical school
Source: PLoS One. 2022 Sep 15;17(9):e0274499. doi: 10.1371/journal.pone.0274499 (PMC9477294; doi:10.1371/journal.pone.0274499)
Supplement: Attachment — Submitted filename: Response to reviewers and editors PLOS One 2022-06-10.docx [file pone.0274499.s006.docx]

## Response to reviewers’ comments

May 26, 2022

To:	Muhammad Shahzad Aslam, Ph.D., M.Phil., Pharm-D

Academic Editor

PLOS ONE

Dear Dr. Muhammad Shahzad Aslam,

Thank you for the opportunity to reply to the reviewers’ comments. The comments and our responses are documented in the following table.

| **Reviewers’ comment** | **Our reply** |
| --- | --- |
| **Reviewer #1** |  |
| Abstract: some of the strongest data, in my opinion, is the aOR of 45 for FPs practicing in northern Ontario who completed UG and PG at NOSM vs. rural background aOR = 5 Would recommend adding this to the abstract. | We have revised the abstract to include the adjusted odds ratios and their 95% confidence intervals. |
| Line 21 “We used a cross-sectional study to measured, …”  Is the word “measured” necessary? It first appears to be a typo (e.g. to measure) | Our thanks for identifying these and other typos.  The abstract was revised to include the above suggestion and the typo was resolved. |
| Line 47-48 “NOSM selects students from underserved and economically deprived regions of Canada with a focus on northern Ontario.”  This can be strengthened with some data, such as “____% of matriculating students at NOSM are from….” | We’ve revised this paragraph to add information on matriculating students. |
| Line 60 typo “with most of the these” | Fixed, thanks. |
| Lines 269-270. “Completing both UG and PG at NOSM had the highest aOR of 45.7.”  Since there is a lot of data, would recommend qualifying this statement, “Completing both UG and PG at NOSM had the highest aOR of 45.7 for FPs practising in northern Ontario.” | We have revised the relevant sentences to read…” FPs who completed both UG and PG at NOSM had highest magnitude adjusted odds ratio (aOR) of practising in northern Ontario relative to those FPs who only completed their UG at NOSM. This aOR of 46 was three-times the magnitude of the aOR for completing just the PG at NOSM and almost nine-times the magnitude for having a northern Ontario background.” |
| Line 280-282 “The overall percentage was nearly twice that of the 49% (508/1047) of doctors who graduated 1973 – 2008 from the Memorial University of Newfoundland (MUN) and did at least some of their PG training at MUN and were practising in MUN’s service region in 2014.[27]”  Intriguing. A very nice comparator. For those not familiar, what is the rationale for using MUN as a comparator? Are they similarly situated? | We have added information on how MUN compares with NOSM. |
| Lines 326-334 seem out of place for this manuscript [“Recent increases in the use of virtual care…”] | Our rationale for including this paragraph is that virtual care offers an alternative to physically locating physicians in regions of need. We argue that, at least, physicians should be trained to care for patients in underserved regions if they are to provide effective virtual care. We also suggest that it is better still to have physicians locate their practice in underserved areas. We have added text to flesh out our arguments. |
| Line 347 It’s fair to say the following, yes? The second, and more influential factor in our study, is to provide undergraduate and postgraduate medical education immersed in the regions, whereby students and trainees are living and learning in the community and in the clinical settings where they are expected to practice in the future. | We added the suggested clause to this sentence. |
| Many are going to be interested in this conclusion because while we recognize the value of admitting students from rural backgrounds into medical school, and we recognize the role of the medical school educational environment, we do not know which is more influential. This study appears to help answer that.  This is an excellent study and most helpful to those who are interested in learning which factors weigh most heavily toward recruitment of primary care into rural and underserved settings. | We thank the reviewer for these kind comments |
| One additional limitation: I understand that Canada manages their residency slots, and this helps produce a higher percentage of family physicians. It may be worth adding this since not all countries will have this in place. | We have added some information to the Methods section on medical education in Canada and on residency matching to provide readers with the necessary context. |
| This certainly adds to the literature on the need for rural/underserved exposure during medical school. If not referenced, consider reviewing some of the recent literature out of Australia that also supports this. | We have added more references including some very recently published studies from Australia. |
| **Reviewer #2** |  |
| L51: Put a reference at the end of the paragraph. | We have revised the paragraph and have included the appropriate reference. |
| L162: Table 1 needs to be re-organized in a better way. | Table 1 has been reorganized. |
| L163: The total calculation should be either in a form of ‘row’ or ‘column’ total, but the authors have calculated the percentages out of the grand total. | We have calculated percentages in the revised Table 1 to best illustrate the point that we wish to make. We used the percentage of the total in the part of the Table that presents rural or urban hometown vs hometown region, in part because we don’t wish to prioritise rurality over region. |
| L170 and downwards: There are a lot of explanations and definitions that can be mentioned in the methods section rather than putting them under the table. | We have added to the existing material in the Methods section and have removed most of the Table notes. |
| L191-196: Table 2 is not accepted in this format. The text contains details like p values and percentages that are not present in the table. The authors should calculate a row percentage and should put the percentage (%) between brackets next to each frequency, and in the last column they should mention the p value. | Table 2 contains detailed data that are collapsed in different ways to test for differences among the three specialty groups in rural-urban practice location or in northern region practice location. The text obtains specific values from Table 2, which are used in these exact tests.  We deliberately presented only the counts in Table 2 to avoid confusion. We suggest that Table 2 would become too cluttered and complex if we were to place the percentages and p-values and mark the desired comparisons in the table itself. |

Responses to editors’ comments appear on the following pages.

## Response to Editors’ comments/Journal requirements

May 26, 2022 (Additional material was added June 10, 2022. Please see the last Table)

The following table documents our response to the itemized Journal requirements.

| **Journal requirements** | **Our Reply (May 26, 2022)** |
| --- | --- |
| 1. “Please ensure that your manuscript meets PLOS ONE's style requirements, including those for file naming.” | Our manuscript meets PLOS ONE's style requirements, including those for file naming. |
| 2. “Please provide additional details regarding participant consent.” | We have added details to the Methods section on informed consent and participant age. |
| 3. [re “competing interests”] Please add “This does not alter our adherence to PLOS ONE policies on sharing data and materials.” | “Sharing materials” does not apply to our study. The modified Competing Interests section now reads:  “JCH works part time for Northern Ontario School of Medicine (NOSM) as a research tutor in the Family Medicine program. RPS is the Founding Dean Emeritus of NOSM. MGF worked for Centre for Rural and Northern Health Research-Laurentian during preparation of the manuscript and since September 2020 has worked full time for NOSM as an Analyst in the Office of Institutional Intelligence. This does not alter the authors’ adherence to PLOS ONE policies on sharing data.” |
| 4. [Re Availability of minimal data set]  NOTE: DOI has been updated since May 26, 2022. | We have generated a minimal data set, which in combination with the detailed tables and supplementary tables will provide data to repeat salient analyses.  Hogenbirk J. Replication data for hometown and practice location of NOSM-trained physicians. Borealis, the Canadian Dataverse Repository (formerly Scholars Portal Dataverse); 2022. Available from: <https://doi.org/10.5683/SP3/70LGLA>  We note that our study collected detailed demographic (e.g., age, gender, linguistic-cultural group) and detailed location data including birthplace of practising physicians and we are limited by the conditions of our ethical approval as to what can be shared. |
| 5. [Re Figure may contain copyrighted images] | Figure 1 was created by a colleague specifically for this manuscript and is available to be published under the Creative Commons Attribution Licence (CC BY 4.0). Our colleague has provided explicit written permission. |
| 6. “Please review your reference list to ensure that it is complete and correct.” | Yes, the list is complete and correct. |

## Response to Editors’ comments received in an email dated June 9, 2022

| **Journal requirements** | **Our Reply (June 10, 2022)** |
| --- | --- |
| 1. [re “competing interests”] Please add “This does not alter our adherence to PLOS ONE policies on sharing data and materials.” | “Sharing materials” does not apply to our study. The modified Competing Interests section now reads:  “JCH works part time for Northern Ontario School of Medicine (NOSM) as a research tutor in the Family Medicine program. RPS is the Founding Dean Emeritus of NOSM. MGF worked for Centre for Rural and Northern Health Research-Laurentian during preparation of the manuscript and since September 2020 has worked full time for NOSM as an Analyst in the Office of Institutional Intelligence. This does not alter the authors’ adherence to PLOS ONE policies on sharing data.”  This text has been added to our revised cover letter. |
| 2. response to reviewers | The first table in this document contains our response to reviewers’ comments. Our detailed response was submitted May 26, 2022. There are no changes to our reply since May 26, 2022. |
| 3. [Re Figure may contain copyrighted images] | a) Figure 1 (map) was created by a colleague using ARCGIS software. The map was specifically created for this manuscript and is available to be published under the Creative Commons Attribution Licence (CC BY 4.0). Our colleague has provided explicit written permission.  b) The map has not been previously copyrighted.  c) To the best of our knowledge, images in the map have not been previously copyrighted. |
| Other item | Please note that the DOI for the shared dataset has changed. The updated DOI is: <https://doi.org/10.5683/SP3/70LGLA> |

Please contact us should you have any further questions.

Respectfully submitted,

John C. Hogenbirk, Roger Strasser, Margaret French
