# Supplementary material for: Ten years of graduates: A cross-sectional study of the practice location of doctors trained at a socially accountable medical school
Source: PLoS One. 2022 Sep 15;17(9):e0274499. doi: 10.1371/journal.pone.0274499 (PMC9477294; doi:10.1371/journal.pone.0274499)
Supplement: Attachment — Submitted filename: Comments_version2.docx [file pone.0274499.s008.docx]

Comments

L51: Put a reference at the end of the paragraph.

L162: Table 1 needs to be re-organized in a better way.

L163: The total calculation should be either in a form of ‘row’ or ‘column’ total, but the authors have calculated the percentages out of the grand total.

L170 and downwards: There are a lot of explanations and definitions that can be mentioned in the methods section rather than putting them under the table.

L191-196: Table 2 is not accepted in this format. The text contains details like p values and percentages that are not present in the table. The authors should calculate a row percentage and should put the percentage (%) between brackets next to each frequency, and in the last column they should mention the p value.

Note: The authors are requested to respond positively to the highlighted note.
